# Supplementary material for: Risk factors for psychiatric symptoms in patients with long COVID: A systematic review
Source: PLoS One. 2023 Apr 7;18(4):e0284075. doi: 10.1371/journal.pone.0284075 (PMC10081737; doi:10.1371/journal.pone.0284075)
Supplement: S1 Table — (DOCX) [file pone.0284075.s002.docx]

**S1 Table. Full non-significant risk factors**

| **Non-significant RF** | **Author** |
| --- | --- |
| **Depression** |  |
| Age | Huarcaya-Victoria et al., 2021 [16]  Menges et al., 2021 [22]  Alemanno et al., 2021 [23]  C. Huang et al., 2020 [26]  Grover et al., 2021 [34]  O’ Sullivan et al., 2021 [36] |
| Sex | Menges et al., 2021 [22]  Grover et al., 2021 [34] |
| Marital status | Huarcaya-Victoria et al., 2021 [16]  Grover et al., 2021 [34] |
| Educational degree | Huarcaya-Victoria et al., 2021 [16] |
| Job status | Huarcaya-Victoria et al., 2021 [16] |
| Profess a religion | Huarcaya-Victoria et al., 2021 [16] |
| Place of origin before hospitalization | Huarcaya-Victoria et al., 2021 [16] |
| Current home | Huarcaya-Victoria et al., 2021 [16] |
| History of family member infected by COVID-19 | Huarcaya-Victoria et al., 2021 [16] |
| Time hospitalized | Huarcaya-Victoria et al., 2021 [16]  Grover et al., 2021 [34] |
| MLR upon admission | Huarcaya-Victoria et al., 2021 [16] |
| Follow-up time | Huarcaya-Victoria et al., 2021 [16] |
| Obesity | Fernández-de-Las-Peñas, Torres-Macho, et al., 2021 [17] |
| Time since diagnosis | Menges et al., 2021 [22] |
| Days since discharge | Grover et al., 2021 [34] |
| Initial symptom severity | Menges et al., 2021 [22] |
| Hospitalization | Menges et al., 2021 [22] |
| ICU admission | Menges et al., 2021 [22] |
| Duration of ICU stay | Grover et al., 2021 [34] |
| Smoking status | Menges et al., 2021 [22] |
| Comorbidity | Menges et al., 2021 [22] |
| Income | Menges et al., 2021 [22] |
| BMI | Menges et al., 2021 [22] |
| Current smoker | C. Huang et al., 2020 [26] |
| Former smoker | C. Huang et al., 2020 [26] |
| College or higher education | C. Huang et al., 2020 [26] |
| Disease severity scale 4 | C. Huang et al., 2020 [26] |
| Disease severity scale 5-6 | C. Huang et al., 2020 [26] |
| Corticosteroid’s administration | C. Huang et al., 2020 [26] |
| Antiviral | C. Huang et al., 2020 [26] |
| Intravenous immunoglobulin | C. Huang et al., 2020 [26] |
| Neurological complication during hospitalization | Frontera et al., 2021 [31] |
| History of stroke | Frontera et al., 2021 [31] |
| Pre-existing migrain | Fernández-de-las-Peñas, Gómez-Mayordomo, Cuadrado, et al., 2021 [33] |
| Presence or absence of physical illness | Grover et al., 2021 [34] |
| Acute SOB | O’ Sullivan et al., 2021 [36] |
| Fever | O’ Sullivan et al., 2021 [36] |
| Fatigue | O’ Sullivan et al., 2021 [36] |
| Cough | O’ Sullivan et al., 2021 [36] |
| **Anxiety** |  |
| Age | Huarcaya-Victoria et al., 2021 [16]  C. Huang et al., 2020 [26]  Frontera et al., 2021 [31]  Grover et al., 2021 [34] |
| Sex | Grover et al., 2021 [34] |
| Marital status | Huarcaya-Victoria et al., 2021 [16]  Grover et al., 2021 [34] |
| Educational degree | Huarcaya-Victoria et al., 2021 [16] |
| Job status | Huarcaya-Victoria et al., 2021 [16] |
| Profess a religion | Huarcaya-Victoria et al., 2021 [16] |
| Place of origin before hospitalization | Huarcaya-Victoria et al., 2021 [16] |
| Current home | Huarcaya-Victoria et al., 2021 [16] |
| Live with | Huarcaya-Victoria et al., 2021 [16] |
| Loss of a family member due to COVID-19 | Huarcaya-Victoria et al., 2021 [16] |
| Self-perception of the COVID-19 severity | Huarcaya-Victoria et al., 2021 [16] |
| Time hospitalized | Huarcaya-Victoria et al., 2021 [16] |
| NLR upon admission | Huarcaya-Victoria et al., 2021 [16] |
| MLR upon admission | Huarcaya-Victoria et al., 2021 [16] |
| Follow up time | Huarcaya-Victoria et al., 2021 [16] |
| Obesity | Fernández-de-Las-Peñas, Torres-Macho, et al., 2021 [17] |
| BMI | Frontera et al., 2021 [31] |
| Hospitalization | Dankowski et al., 2021 [20] |
| Current smoker | C. Huang et al., 2020 [26] |
| Former smoker | C. Huang et al., 2020 [26] |
| College or higher education | C. Huang et al., 2020 [26] |
| Comorbidity | C. Huang et al., 2020 [26] |
| Disease severity scale 4 | C. Huang et al., 2020 [26] |
| Disease severity scale 5-6 | C. Huang et al., 2020 [26] |
| Corticosteroid’s administration | C. Huang et al., 2020 [26] |
| Antiviral | C. Huang et al., 2020 [26] |
| Intravenous immunoglobulin | C. Huang et al., 2020 [26] |
| Neurological complication during hospitalization | Frontera et al., 2021 [31] |
| History of dementia | Frontera et al., 2021 [31] |
| Pre-existing migrain | Fernández-de-las-Peñas, Gómez-Mayordomo, Cuadrado, et al., 2021 [33] |
| Presence or absence of physical illness | Grover et al., 2021 [34] |
| Duration of hodpital stay | Grover et al., 2021 [34] |
| Duration of ICU stay | Grover et al., 2021 [34] |
| Days since discharge | Grover et al., 2021 [34] |
| **PTSD** |  |
| Gender | Huarcaya-Victoria et al., 2021 [16]  De Lorenzo et al., 2021 [29]  Grover et al., 2021 [34] |
| Age | Huarcaya-Victoria et al., 2021 [16]  De Lorenzo et al., 2021 [29]  Grover et al., 2021 [34] |
| Marital status | Huarcaya-Victoria et al., 2021 [16]  Grover et al., 2021 [34] |
| Educational degree | Huarcaya-Victoria et al., 2021 [16] |
| Job status | Huarcaya-Victoria et al., 2021 [16] |
| Profess a religion | Huarcaya-Victoria et al., 2021 [16] |
| Place of origin before hospitalization | Huarcaya-Victoria et al., 2021 [16] |
| Current home | Huarcaya-Victoria et al., 2021 [16] |
| Live with | Huarcaya-Victoria et al., 2021 [16] |
| History of family member infected by COVID-19 | Huarcaya-Victoria et al., 2021 [16] |
| Loss of a family member due to the COVID-19 | Huarcaya-Victoria et al., 2021 [16] |
| Time hospitalized | Huarcaya-Victoria et al., 2021 [16] |
| NLR upon admission | Huarcaya-Victoria et al., 2021 [16] |
| MLR upon admission | Huarcaya-Victoria et al., 2021 [16] |
| Ethnicity | De Lorenzo et al., 2021 [29] |
| BMI | De Lorenzo et al., 2021 [29] |
| Comorbidities | De Lorenzo et al., 2021 [29] |
| Hospitalization | De Lorenzo et al., 2021 [29] |
| Length of stay | De Lorenzo et al., 2021 [29]  Grover et al., 2021 [34] |
| Duration of ICU stay | Grover et al., 2021 [34] |
| Non invasive ventilation | De Lorenzo et al., 2021 [29] |
| Presence or absence of physical illness | Grover et al., 2021 [34] |
| Days since discharge | Grover et al., 2021 [34] |
| **Sleep disturbances, poor sleep quality and insomnia** |  |
| Sex | Romero-Duarte et al., 2021 [24] |
| Age over 65 years | Shang et al., 2021 [30] |
| Severity | Shang et al., 2021 [30] |
| ICU admission | Shang et al., 2021 [30]  Frontera et al., 2021 [31] |
| Intubation | Shang et al., 2021 [30] |
| In hospital days | Shang et al., 2021 [30]  Frontera et al., 2021 [31] |
| Neurological complication during hospitalization | Frontera et al., 2021 [31] |
| Corticosteroid use | Frontera et al., 2021 [31] |
| Anticoagulant use | Frontera et al., 2021 [31] |
| Pre-existing migrain | Fernández-de-las-Peñas, Gómez-Mayordomo, Cuadrado, et al., 2021 [33] |
| **Cognitive deficits** |  |
| Sex | Gouraud et al., 2021 [35]  Grover et al., 2021 [34] |
| Gender | Grover et al., 2021 [34] |
| Marital status | Grover et al., 2021 [34] |
| Neurological complication during hospitalization | Frontera et al., 2021 [31] |
| Presence or absence of physical illness | Grover et al., 2021 [34] |
| Duration of hospital stay | Grover et al., 2021 [34] |
| Duration of ICU stay | Grover et al., 2021 [34] |
| Days since discharge | Grover et al., 2021 [34] |
