# Supplementary material for: Risk factors for psychiatric symptoms in patients with long COVID: A systematic review
Source: PLoS One. 2023 Apr 7;18(4):e0284075. doi: 10.1371/journal.pone.0284075 (PMC10081737; doi:10.1371/journal.pone.0284075)
Supplement: Attachment — Submitted filename: Response to Reviewers.docx [file pone.0284075.s005.docx]

**Rebuttal Letter**

Dear editor and reviewer,

We thank the editor and reviewer for your valuable comments and suggestions. We carefully reviewed your comments/suggestions and revised the manuscript accordingly.

Revisions are highlighted using the yellow color in the text.

Journal requirements:

When submitting your revision, we need you to address these additional requirements.

1. Please ensure that your manuscript meets PLOS ONE's style requirements, including those for file naming. The PLOS ONE style templates can be found at

https://journals.plos.org/plosone/s/file?id=wjVg/PLOSOne_formatting_sample_main_body.pdf and

<https://journals.plos.org/plosone/s/file?id=ba62/PLOSOne_formatting_sample_title_authors_affiliations.pdf>".

[Note: HTML markup is below. Please do not edit.]

- Thank you very much for the input. We have revised the manuscript according to PLOS ONE’s style requirements and templates, according to your suggestion.

Reviewers' comments:

**Comments to the Author**

Review Comments to the Author

Reviewer #1: Major comments: This is an interesting systematic review about risk factors of psychiatric symptoms in patients with long COVID.
However, some point should be improved. The English language should be checked by a native English speaker. Also, the authors should be more precise especially when citing references, because in the introduction section many citations don’t match the purpose for which they are cited.

- Thank you very much for your consideration and advice. We had the manuscript checked and edited by our academic proofreader (certificate attached). We also carefully re-checked and revised the citations in our manuscript.

Specific comments

Introduction section

Page 3:
Please correct your statement about reference 6, since it is not dealing with COVID-19 participants, but with general population during COVID-19, which is very different from what you are dealing in the following sentence about reference 7.

- Thank you very much for your input, we have changed the references and statement in order to provide more clarity on the topic. Changes were made on page 3, line 44-46.

Lines 44-46 now read: “A systematic review in 2021 showed that depression, anxiety, post-traumatic stress disorder (PTSD), cognitive deficits, fatigue and sleep disturbances were commonly found in COVID-19 survivors [6]”

Page 4: again, the citation of reference 9 is false, this reference is A meta-analysis dealing with acute psychiatric signs of coronaviruses and not persistence after recovery.

- Thank you very much for your correction, we have changed the reference 9. We have added “A preliminary data suggested that some psychiatric disorder such as anxiety and depression persisted in patients who had already recovered from COVID-19 [19].” on page 3, line 58-59.

Results section:

The term “somatic symptoms” is unclear to me, since only one study has evaluated this dimension, some more data about the nature of “somatic symptoms” may be given by the author for informative purpose.

- We have added more information about somatic symptoms in line 255-257.

Lines 255-257 now read: “Somatic symptoms were assessed using PHQ-15 [17]. It evaluated physical symptoms experienced by the patients, including stomach pain, back pain, headaches, and other physical symptoms.”

In each section, you should not cite the unsignificant risk factors for each pathology. They should be mentioned in your table S1 and not in the text. It is better to focus on positive results and contradictory results between studies.

- Thank you. We have erased statements about unsignificant risk factors, and listed them in Table S1, following your suggestion.

Discussion section

This section is too redundant compared to the result section. You should not repeat the entire results, but rather discuss more deeply some points, maybe raising pathophysiological hypothesis. You should also stress on what is new in your study since the last systematic review on the subject published in October 2021

- We’ve revised the discussion section according to your suggestion. We’ve also added some patophysiological hypothesis in the discussion section.

Lines 301-304 now read: “Anxiety is the most common psychiatric symptoms seen in Long COVID individuals in our study. Psychiatric complications due to COVID-19 could happen both by immune response to the coronavirus, or due to psychological stressors during pandemic such as social isolation, concerns about infecting others, and stigma [9]”

Lines 308-311 now read: “Seens et al., stated that this happens due to the feminine tendency in mental health toward internalizing disorders. In addition, women are more accustomed to interactions and social support outside of the household for maintaining mental health, therefore, social isolation might have a negative impact on female gender [42]”

Lines 318-321 now read: “A meta-analysis showed that inflammatory cytokines such as interleukin-6, TNF-alpha, and interleukin-10 were positively associated with depression, studies further showed that those cytokines were associated with post COVID sequelae and those inflammatory cytokines stays elevated even until 8 months post COVID infection [43, 44]”

Figure 1: please improve the quality of the figure, I can’t read it in its current state.

- Thank you for the input, we have improved the quality of the figure according to your input. We also have used PACE provided by the PLOS ONE journal to suit the format of the figure.
