# Supplementary material for: Risk factors for psychiatric symptoms in patients with long COVID: A systematic review
Source: PLoS One. 2023 Apr 7;18(4):e0284075. doi: 10.1371/journal.pone.0284075 (PMC10081737; doi:10.1371/journal.pone.0284075)
Supplement: Attachment — Submitted filename: Response to Reviewers.docx [file pone.0284075.s007.docx]

**Rebuttal Letter**

Dear editor and reviewer,

We thank the editor and reviewer for your valuable comments and suggestions. We carefully reviewed your comments/suggestions and revised the manuscript accordingly.

Revisions are tracked using track changes feature of the Microsoft Word.

Journal requirements:

Please include the following items when submitting your revised manuscript:

1. A rebuttal letter that responds to each point raised by the academic editor and reviewer(s). You should upload this letter as a separate file labeled 'Response to Reviewers'.
2. A marked-up copy of your manuscript that highlights changes made to the original version. You should upload this as a separate file labeled 'Revised Manuscript with Track Changes'.
3. An unmarked version of your revised paper without tracked changes. You should upload this as a separate file labeled 'Manuscript'.
4. If you would like to make changes to your financial disclosure, please include your updated statement in your cover letter. Guidelines for resubmitting your figure files are available below the reviewer comments at the end of this letter.

**Comments to the Author**

1. Some grammatical error still present.

**Response**: Thank you for your comment. We have carefully corrected the grammatical error.

2. Table 2 is not standard for reporting the systematic review. Please use common ways to report the outcome.

**Response**: Thank you for pointing this out. We have updated the table to more desirable form.

3. Please follow PRISMA guideline.

**Response:** Thank you for your kind reminder. We have followed PRISMA guideline on writing this review. An additional file of PRISMA guideline checklist is attached in this resubmission.

4. The prevalence of psychiatric symptoms are vary between studies. Please try to explore the causes. Is it because of different diagnosis tools? Is it because of different patient characteristics ?

**Response:** Thank you for your input, we have added more information of the explanation in wide range of prevalence found in our review. Changes were made on page 24-25, line 356-359.

“Furthermore, we found a wide range of prevalence of psychiatric symptoms between studies. The use of different instruments to assess the symptoms was potentially a major huge factor of heterogeneity in the prevalence. In addition, the difference in follow-up times between studies was a contributor in the prevalence variation.”
