# Supplementary material for: Risk factors for psychiatric symptoms in patients with long COVID: A systematic review
Source: PLoS One. 2023 Apr 7;18(4):e0284075. doi: 10.1371/journal.pone.0284075 (PMC10081737; doi:10.1371/journal.pone.0284075)
Supplement: Attachment — Submitted filename: Response to Reviewers.docx [file pone.0284075.s008.docx]

**Rebuttal Letter**

Dear editor and reviewer,

We thank the editor and reviewer for your valuable comments and suggestions. We carefully reviewed your comments/suggestions and revised the manuscript accordingly.

Revisions are tracked using track changes feature of the Microsoft Word.

Journal requirements:

Please include the following items when submitting your revised manuscript:

- A rebuttal letter that responds to each point raised by the academic editor and reviewer(s). You should upload this letter as a separate file labeled 'Response to Reviewers'.
- A marked-up copy of your manuscript that highlights changes made to the original version. You should upload this as a separate file labeled 'Revised Manuscript with Track Changes'.
- An unmarked version of your revised paper without tracked changes. You should upload this as a separate file labeled 'Manuscript'.

**Comments to the Author**

1. If the authors have adequately addressed your comments raised in a previous round of review and you feel that this manuscript is now acceptable for publication, you may indicate that here to bypass the “Comments to the Author” section, enter your conflict-of-interest statement in the “Confidential to Editor” section, and submit your "Accept" recommendation.

Reviewer #1: All comments have been addressed

Reviewer #2: All comments have been addressed

**Response**: Thank you for your response.

2. Is the manuscript technically sound, and do the data support the conclusions?

The manuscript must describe a technically sound piece of scientific research with data that supports the conclusions. Experiments must have been conducted rigorously, with appropriate controls, replication, and sample sizes. The conclusions must be drawn appropriately based on the data presented.

Reviewer #1: Yes

Reviewer #2: Yes

**Response**: Thank you for your response.

3. Has the statistical analysis been performed appropriately and rigorously?

Reviewer #1: Yes

Reviewer #2: Yes

**Response**: Thank you for your response.

4. Have the authors made all data underlying the findings in their manuscript fully available?

The PLOS Data policy requires authors to make all data underlying the findings described in their manuscript fully available without restriction, with rare exception (please refer to the Data Availability Statement in the manuscript PDF file). The data should be provided as part of the manuscript or its supporting information, or deposited to a public repository. For example, in addition to summary statistics, the data points behind means, medians and variance measures should be available. If there are restrictions on publicly sharing data—e.g. participant privacy or use of data from a third party—those must be specified.

Reviewer #1: Yes

Reviewer #2: Yes

**Response**: Thank you for your response.

5. Is the manuscript presented in an intelligible fashion and written in standard English?

PLOS ONE does not copyedit accepted manuscripts, so the language in submitted articles must be clear, correct, and unambiguous. Any typographical or grammatical errors should be corrected at revision, so please note any specific errors here.

Reviewer #1: Yes

Reviewer #2: Yes

**Response**: Thank you for your response.

6. Review Comments to the Author

Please use the space provided to explain your answers to the questions above. You may also include additional comments for the author, including concerns about dual publication, research ethics, or publication ethics. (Please upload your review as an attachment if it exceeds 20,000 characters)

Reviewer #1: The quality of the MS has significantly improved. The authors provided supplementary materials with available data. The English has also been revised.

Reviewer #2: The figure quality of PRISMA should be improved. Please revise table 2. It is too long. You should mention "risk factors" not statistical analysis for independent risk factors. Begin the discussion with the finding of your review.

**Response**: Thank you for your response. We have increased the quality of the PRISMA flow chart figure. We have shortened the Table 2 (we remove the p-value). The discussion session now is started with the mind finding of this review (pg. 18 page 286-287)

Note:

- We do not deposit our laboratory/study protocols in protocols.io because we have registered our review in PROSPERO.
- We have uploaded your figure files to the Preflight Analysis and Conversion Engine (PACE) digital diagnostic tool.
- We have added three new paragraphs. The first one discusses the pathophysiology of Long COVID (pg 20, ln 320-ln 333). The second one discusses the bidirectionality of physical and mental health of Long COVID (pg 20, ln 334-pg 21, ln 338). The third one discusses the burden of the Long COVID (pg 21, ln 339-346).
